# Silencing and Transcriptional Regulation of Endogenous Retroviruses: An Overview

**DOI:** 10.3390/v12080884

**Published:** 2020-08-13

**Authors:** Franziska K. Geis, Stephen P. Goff

**Affiliations:** 1 Department of Biochemistry and Molecular Biophysics, Columbia University Medical Center, New York, NY 10032, USA; fkg2105@cumc.columbia.edu; 2 Department of Microbiology and Immunology, Columbia University Medical Center, New York, NY 10032, USA; 3 Howard Hughes Medical Institute, Columbia University Medical Center, New York, NY 10032, USA

**Keywords:** endogenous retroviruses (ERV), transposable elements, transcriptional silencing, ERV expression and diseases, co-option of ERV functions

## Abstract

Almost half of the human genome is made up of transposable elements (TEs), and about 8% consists of endogenous retroviruses (ERVs). ERVs are remnants of ancient exogenous retrovirus infections of the germ line. Most TEs are inactive and not detrimental to the host. They are tightly regulated to ensure genomic stability of the host and avoid deregulation of nearby gene loci. Histone-based posttranslational modifications such as H3K9 trimethylation are one of the main silencing mechanisms. Trim28 is one of the identified master regulators of silencing, which recruits most prominently the H3K9 methyltransferase Setdb1, among other factors. Sumoylation and ATP-dependent chromatin remodeling factors seem to contribute to proper localization of Trim28 to ERV sequences and promote Trim28 interaction with Setdb1. Additionally, DNA methylation as well as RNA-mediated targeting of TEs such as piRNA-based silencing play important roles in ERV regulation. Despite the involvement of ERV overexpression in several cancer types, autoimmune diseases, and viral pathologies, ERVs are now also appreciated for their potential positive role in evolution. ERVs can provide new regulatory gene elements or novel binding sites for transcription factors, and ERV gene products can even be repurposed for the benefit of the host.

## 1. Introduction

Transposable elements (TEs) are a large component of all eukaryote genomes, comprising a major fraction of all their repetitive sequences. As much as 40–60% of the mammalian genome consists of TEs [1,2,3]. Importantly, TEs are not randomly dispersed in the genome. Their distribution is determined by TE integration site preferences, but also by selective forces operating on the phenotypes resulting from the insertion [4]. The sequences serving as binding sites for transcription factors and the various determinants of the chromatin state present on the TEs all contribute to the final distribution throughout the genome over evolutionary times [5]. In order to permanently persist in the host, TEs have achieved an efficiency of propagation, giving an appropriate balance of disadvantageous and advantageous consequences for the host [4].

TEs can be categorized into two classes: DNA transposons and retrotransposons. The retrotransposons are further subdivided by the absence or presence of long terminal repeats (LTRs) flanking a central coding region. Prominent non-LTR retrotransposons are the long interspersed nuclear elements (LINEs), or the short interspersed nuclear elements (SINEs)

**Figure 1:**
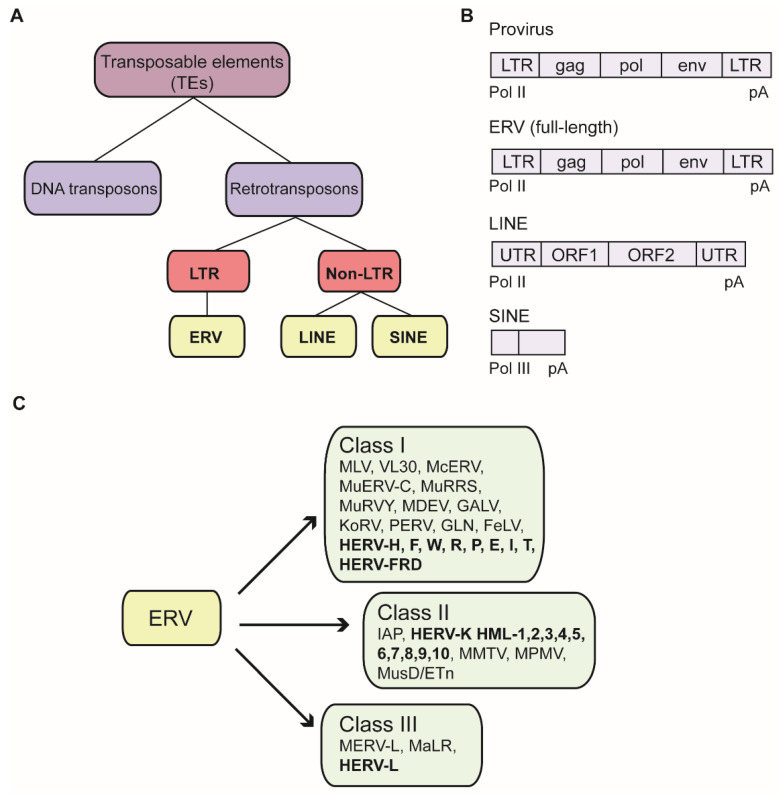
Organization, structure and classification of transposable elements (TEs). (**A**) TEs can be divided into DNA transposons or retrotransposons. Retrotransposons are defined either as long-terminal repeat (LTR)-retrotransposons, such as endogenous retroviruses (ERVs) or non- LTR retrotransposons, like long interspersed elements (LINE) or short interspersed elements (SINE). (**B**) Genomic structures are shown for provirus, ERV, LINE and SINE. A full-lenth version of ERV is shown exemplary, but genomic organizations can vary. Abbreviations: Pol II: polymerase III; pA: poly (A) tail; UTR: untranslated region; ORF: open reading frame. (**C**) Classification of ERVs comprises three classes. Human ERVs are depicted in bold.

Retrotransposons flanked by LTRs share high similarities with exogenous retroviral proviruses and are named endogenous retroviruses (ERVs), which are the remnants of ancient virus infections (Figure 1A) [6]. These endogenized forms of viral sequences become established after germ cell infections by exogenous retroviruses. Once successfully integrated into the germ line genome, proviruses are transmitted vertically by standard Mendelian inheritance. The oldest known human ERV family (HERV-L) is probably more than 60 to 70 million years old, whereas the youngest element of the HERV-K family (HML-2) is approximately five million years old [7]. During the tremendous amount of time between their introduction into the germ line and the present, the vast majority of ERVs have accumulated disruptive mutations and have thereby lost their ability to produce virus or even to retrotranspose within the genome. An exogenous retrovirus genome typically consists of a common set of at least four genes: *gag*, which encodes structural matrix and capsid proteins; *pro*, encoding the viral protease; *pol*, which encodes the retroviral enzymes reverse transcriptase as well as integrase; and *env*, encoding glycoproteins that determine host cell tropism (Figure 1B). Due to accumulated mutations, ERV sequences have preserved the features of their original provirus to a highly variable extent, ranging from the retention of a complete set of LTRs and retroviral genes to the retention of only highly fragmented portions of the parental virus genomes. Many ERV sequences consist only of solitary LTRs, which are most likely generated by homologous recombination between the 5′ and 3′ LTRs [8].

It has been known since the mid-1950s that TEs are highly regulated, with the discovery of “controlling elements” in maize [9]. However, it took another three decades until the molecular structure of the mobile elements, the mechanism of their transposition, and the means by which they can be regulated could be understood. The similarity of ERVs to retroviruses could only be appreciated with the identification of the enzyme reverse transcriptase [10,11] and an understanding of the retroviral life cycle. ERVs were considered by many as not likely to be useful to the cell and were often described as “junk DNA”, “viral hitchhikers”, and “fossil viruses” [12]. At best, ERVs were assumed to be able to teach us about the existence and the nature of ancient extinct viruses as well as our own evolutionary history, since these fossil signatures are witnesses of events from our past [8]. ERV functions were soon found to have major consequences for the host in the development of many diseases. Furthermore, over the years, it has become apparent that ERVs can provide important useful functions, and the phenomenon of the co-option of ERV genes or products has become more clear. Whole-genome sequencing technology, starting about 20 years ago, has accelerated the identification and phylogenetic investigation of ERVs. One of many prominent topics in the field of ERVs is the transcriptional regulation and control of these ancient viral remnants where the regulation of ERV expression was proven to be more dynamic and influential than initially assumed.

In this review, we will summarize recent findings concerning the silencing and transcriptional regulation of ERVs. Furthermore, we will give an overview of how ERVs fulfill physiological functions and can be utilized as targets for cancer therapies. This review particularly focuses on the silencing and transcriptional regulation of ERVs. However, some regulatory mechanisms have been described solely or additionally for non-LTR retrotransposons and are therefore included briefly in this article, since they might also impact the transcriptional regulation of ERVs. Due to the considerable differences between mouse and human ERVs, we discuss the elements in the two species in this review separately. Importantly, the majority of findings regarding ERV silencing has been studied in the murine system, mostly during embryonic development and in germ cells. Many mechanisms and concepts of mouse ERV regulation might be applicable to human ERVs, but there may be considerable differences.

## 2. Epigenetic Silencing of Murine ERVs

### 2.1. Nomenclature of Murine ERVs

As the number of identified ERV sequences has grown, it has become more and more challenging to establish and maintain a systematic and common ERV nomenclature. Generally, ERVs have been classified by their relationship to exogenous retroviruses, which comprise seven genera [13,14]. For this purpose, taxonomic annotation largely follows the International Committee for Virus Taxonomy (ICTV). Conventionally, ERVs are grouped into three classes, based on the sequence similarity of their *pol* regions with reverse transcriptase sequences of exogenous retroviruses: class I resembles gamma- and epsilonretroviruses, class II resemble alpha-, beta- and deltaretroviruses, and class III resemble the spumaviruses [15]. Although mouse and human ERVs share the same nomenclature of three classes based on their *pol* sequences, they differ substantially in their sequences and mobility. The diverse paths of evolution account mainly for this divergence. Mouse representatives of the three ERV classes are those similar to the classical murine leukemia viruses (MLVs) and the virus-like 30S RNA (VL30) elements (class I); those similar to the mouse mammary tumor viruses (MMTV), the MusD family, and the large intracisternal A-particle (IAP) superfamily with about 1000 copies/cell (class II); and the MERV-L family (class III) (Figure 1C) [16]. Recently, a novel nomenclature has been proposed to establish a more unified system that provides information of the ERV group, the genomic loci as a unique numeric ID, and the species. This design allows for the integration of the increasing number of new identified ERVs and other TEs into existing schemes [13].

### 2.2. Posttranslational Histone Modifications as a Driving Force in Heterochromatin Formation

Astonishingly, 40% of the mouse genome comprises TEs and 10% of the genome are classified as ERVs. Some of these ERVs in specific mouse lineages are still expressed and are able to replicate [16]. Notably, transcriptional expression of ERVs and the induction of that expression were first described in the early 70s [17]. IAP elements are very abundant and still active, and they are considered as among the most active TEs. IAPs are thought to be responsible for 10% of all spontaneous mutations in the mouse genome [18]. It became quite obvious that transcriptional expression and transposition of ERVs need to be tightly regulated to provide genomic stability and avoid aberrant expression of neighboring genes. Retrotransposition in germ cells would lead to germline mutagenesis and vertical transmission [18,19], whereas mutagenesis in somatic cells could cause oncogenic transformation [20,21]. Our current knowledge of key players inducing transcriptional silencing of ERVs was largely obtained from studies of the silencing mechanisms of exogenous retroviruses [22]. Transcriptional silencing is achieved through the establishment of a heterochromatic structure, which maintains an inactive state of chromatin. The formation of a condensed high-order conformation prevents access to transcriptional machinery and consequently suppresses transcriptional expression [23]. Embryonic stem cells (ESCs) are often utilized as a cell model to study transcriptional regulation of ERVs since the pluripotent cell type is long known for its repression of both exogenous and endogenous retroviruses [24,25,26]. ESCs and their developmental correlates are unique for the global re-writing of the chromatin state of their genomes (e.g. establishment of heterochromatin through *de novo* methylation [27]).

**Figure 2:**
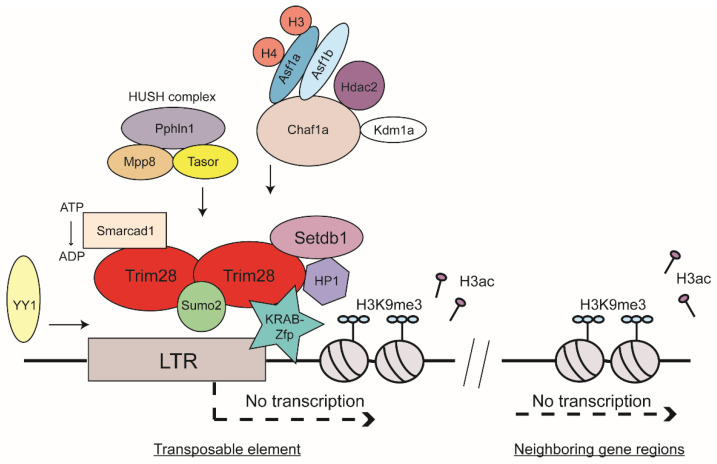
Histone-based silencing of mouse TEs. KRAB-zinc finger proteins (Zfps) bind to TEs, either to the primer binding site or at other specific binding sites in TE sequences, and recruit Trim28. Trim28 is sumoylated via Sumo2, which enhances Trim28 localization to TEs. The chromatin remodeler Smarcad1 uses its ATPase activity to additionally improve Trim28 affinity to TEs and interaction with Setdb1. Setdb1, a H3K9-specific methyltransferase, establishes heterochromatin by formation of the histone H3K9 trimethylation mark, and HP1 binds that mark. H3K9 trimethylation-based silencing can spread to neighboring gene regions. YY1 binds to LTR regions of TEs and promotes TRIM28 recruitment. The HUSH complex can induce silencing through interaction with Trim28/Setdb1 and formation of H3K9 trimethylation. The histone chaperone Chaf1a interacts with Kdm1a and Hdac2 to suppress transcription of class III ERVs and Chaf1 cooprerates with Trim28 to establish H3K9 trimethylation for silencing class I and II ERVs. The histone chaperone isoforms Asf1a and Asf1b help localizing Chaf1a to TEs.

Heterochromatin formation is associated with several observable events including specific posttranslational histone modifications and DNA methylation. These can be induced by specific proteins or RNA-mediated targeting. Characteristic histone marks of heterochromatic regions in the genome are, for example, H3K9, H3K27, or H4K20 methylations [28,29]. Genome-wide analyses revealed enrichment of repressive histone marks such as trimethylation of H3K9 or H4K20 on mouse ERVs [30,31]. H3K9 trimethylation (H3K9me3) has especially been found to be associated with inactive viral chromatin of a broad range of virus DNAs: the unintegrated and integrated forms of exogenous retroviruses as well as ERVs [30,31,32,33,34]. The histone mark H3K9me3 is generated by specific methyltransferases. There have been at least five H3K9-specific methyltransferases described in mammals: Suv39h1, Suv39h2, Setdb1, Glp, and G9a [35]. Knock-out experiments of several methyltransferases have shed light on their involvement in ERV silencing. G9a and Glp have been shown to be required for silencing of MERV-L, a member of class III ERVs, very early in development during the two-cell stage [36,37]. Furthermore, knockout of G9a and Glp led to DNA demethylation of class I and II ERVs. Knock-out of another H3K9 methyltransferase, Suv39h, exhibited reduced H3K9me3 spreading on intact ERVs as well as overall reduced silencing of non-LTR retrotransposon LINE-1 [38]. Probably the most prominent H3K9-specific methyltransferase is Setdb1 (also known as Eset) [39]. Knock-out of Setdb1 results in derepression of ERV classes I and II in murine ESCs [40,41]. Notably, DNA methylation seems to be nonessential for ERV silencing in settings where Setdb1 function is induced and H3K9me3 is established [40,41]. In differentiated somatic cell types, a more distinct role has been recently shown for Setdb1 [42]. Depletion of Setdb1 in different types of somatic cells showed reactivation of specific subsets of ERVs such as VL30 in mouse embryonic fibroblasts [42]. This derepression showed strong dependence on cell-type specific transcription factors. Importantly, the most prominent binding partner of Setdb1 is Trim28 (Figure 2) [43].

### 2.3. Trim28 as a Master Regulator of ERV Silencing

A frequent target of ERV silencing is the so-called primer binding site (PBS), an essential sequence used for the initiation of reverse transcription by a host tRNA serving as primer. Trim28 (also known as Kap1 or Tif1β) is involved in both PBS-dependent and PBS-independent proviral silencing mechanisms. Trim28 is expressed in most cell types, and its level is especially high during early development and in ESCs as well as both in the developing and adult brain [44,45,46]. Trim28 was identified as a universal corepressor protein binding to the Krüppel-associated box (KRAB) domain of KRAB-zinc finger proteins (Zfps) (Figure 2) [47]. Briefly, Zfps are one of the largest protein families in eukaryotes and are extremely diverse in their functions as well as in their structure [48]. Those containing KRAB domains, the KRAB-Zfps, are the largest family of transcriptional regulators in higher vertebrates and are about 420 million years old [49]. Zfps contain an array of a variable number of tandem copies of C2H2 zinc fingers, which confer high DNA-binding specificity. Many of the genes for zinc finger proteins are organized in clusters, and these are thought to be more species-specific compared to zing finger genes outside the cluster, which are considered to be more ancient [49].

Some of the evolutionarily older KRAB-Zfps might even restrict retroviruses on a non-transcriptional level. The so-called SCAN domain of KRAB-Zfps resembles the C-terminal capsid of the human immunodeficiency virus type 1 (HIV-1) [50]. The multimerization phenotype of SCAN also shares similarities with HIV-1 capsid formation [51,52,53], suggesting that these Zfps could affect virion assembly. Some KRAB-Zfps have been reported to bind RNA and proteins, which suggest an additional function beside DNA-binding-mediated mechanisms [53]. Furthermore, evolutionarily young KRAB-Zfps in mice were found to bind currently active ERVs such as IAPs or ETns [54]. The authors suggested that recently active TEs are continuing to shape the expansion and evolution of KRAB-Zfp genes. The so called ‘arms race’ between KRAB-Zfps and TEs is considered as a main driving force for evolution in mammals and is even thought to promote ERV domestication [55].

Sequence-specific DNA binding by the KRAB-Zfps is mediated through the zinc fingers themselves, whereas the KRAB domain recruits corepressors and other chromatin modifiers to induce transcriptional suppression. Most KRAB-Zfps interact with Trim28 [53]. An example of a prominent KRAB-Zpf that binds Trim28 is Zfp809, which binds specifically to the tRNA proline PBS of MLV and mediates silencing of both exogenous and endogenous viral expression in mouse ESCs [56,57,58]. Another example is the product of the *sgp3* gene, which similarly binds specifically to the tRNA glutamine PBS of endogenous MLVs and mediates their silencing in various mouse strains [59]. A novel recently discovered KRAB-Zfp is Zfp708, which also recruits Trim28. Zfp708 specifically binds a member of the ERV-K family and is shown to play an important role during embryonic development through regional formation of epigenetic marks of heterochromatin during epigenetic reprogramming [60]. Another example is the Zfp Yin Yang 1 (YY1), which binds to the LTR-region of many retroviruses, both exogenous and endogenous, and thereby induces silencing by recruitment of Trim28 and its downstream machinery (Figure 2) [61,62,63,64,65]. Importantly, Trim28 was shown to co-localize with heterochromatin protein 1 (HP1) [66], another important key player in heterochromatin formation. It has been shown that the methylation of H3K9 forms a binding site with high affinity for HP1 and therefore the H3K9me3 mark itself recruits HP1 proteins efficiently [67,68,69,70]. Trim28, Setdb1, H3K9me3, and HP1 were all found to be enriched at promoter sequences of genes silenced by the KRAB-Zfp and Trim28 complex (Figure 2) [43]. Trim28 knock-out leads to upregulation of several ERV classes (e.g., IAP and MERV-L [45]). Furthermore, a knock-down of Trim28 led to activation of genes in near proximity to ERVs [71,72].

Trim28 and Setdb1 together seem to regulate ERVs to a different extent than Trim28 alone. Setdb1 knock-out results in derepression of additional ERV classes beyond those seen with single knock-out of Trim28 [40,41,45]. For example, MERV-L expression was exclusively elevated by Trim28 depletion [37]. Recently, a study identified another novel interaction partner of Trim28. The O-linked beta-N-acetylglucosamine transferase (OGT) and Trim28 have been shown to co-localize at methylated promoter regions of ERVs [73]. O-linked glycosylation is a newly described chromatin mark, which is suggested to play a crucial role in transcriptional silencing of ERVs [73].

### 2.4. Sumoylation of Trim28 and HUSH Complex as Contributors to TE Silencing

It has been shown that the sumo pathway plays a significant role in ERV silencing [6,74]. Mammals exhibit four members of the Sumo family: Sumo1, Sumo2, Sumo3, and Sumo4 [75]. Interestingly, sumoylation of Trim28 improved its interaction with Setdb1 and seemed to play a role in providing the proper localization of Trim28 to ERVs [74,76,77]. Sumo2, in contrast to Sumo1 or Sumo3, was identified as having a distinctive role in proviral silencing [74]. Sumo2 knock-down was found to abolish binding of Trim28 at LTRs. Therefore, it has been proposed that Trim28 is sumoylated by Sumo2, which mediates recruitment of Trim28 to the proviral DNA, which then recruits Setdb1 to mediate H3K9me3 formation and thus induces silencing (Figure 2). Another interaction partner of Trim28 was found to be the human silencing hub (HUSH) complex, composed of Tasor (also known as Fam208a), Mpp8 (Mphosph8), and Pphln1 (Periphilin 1). The HUSH complex is enriched at genomic loci associated with H3K9me3 and was reported to interact with Setdb1 as well as MORC2 [39,78,79,80,81,82]. Trim28 together with the HUSH complex component Tasor has been shown to mainly silence evolutionarily young genes enriched in non-LTR retrotransposons in regions rich in LINE-1 elements (Figure 2) [83]. Furthermore, the HUSH complex was shown to contribute to position-effect variegation by its function to spread chromatin states into gene regions [79].

### 2.5. Chromatin Spreading and ATP-Dependent Chromatin Remodeler

The ability of the silencing machinery to act over long distances is one aspect of the mechanisms to ensure and maintain genomic stability. KRAB-Zfps and their corepressor Trim28 are able to suppress activity of promoters several tens of kilobases distant from their DNA-binding sites. This may be achieved by looping across long DNA lengths, but may also involve spreading of heterochromatic regions along DNA. Heterochromatin features include a decrease of H3-acetylation, an increase of H3K9me3, and a reduced level of RNA Pol II recruitment (Figure 2) [84]. Heterochromatin spreading is thought to be less frequently initiated at IAP elements, but is thought to have an effect on the expression of specific genes [85].

Many regulators of chromatin structure may regulate ERV expression, for example through the interactions of chromatin remodelers with Trim28. Recently, it has been shown that the SWI/SNF-like adenosine triphosphate (ATP)-dependent chromatin remodeler Smarcad1 plays a role in ERV regulation in mouse ESC [86]. Depletion of Smarcad1 resulted in upregulation of IAPs and genes in their near vicinity. The SWI/SNF subfamily is known to promote chromatin access by moving nucleosomes and are described to either activate or repress transcription [87]. Smarcad1 was shown to bind Trim28 in mouse ESCs [88]. A genome-wide profile demonstrated that Smarcad1 was enriched at class I and II ERVs, especially at IAPs. Smarcad1 binding to TEs required the presence of Trim28 and also required its ATPase activity (Figure 2). A Smarcad1 ATPase mutant did not alter its binding to Trim28, but overall occupancy of Smarcad1 and Trim28 at IAPs was decreased in the absence of the ATPase function. The authors hypothesized that Zfps, Trim28, and Smarcad1 bind to ERV sequences and induce silencing of ERV expression. In this complex, the ATPase function of Smarcad1 might play a role to stabilize Trim28 binding to ERV. In the absence of Smarcad1, binding to ERV sequences and recruitment of Setdb1 were found to be compromised [86]. Another candidate found to be involved in ERV silencing is Kdm1a (also known as Lsd1), a lysine-specific demethylase. High-throughput mRNA sequencing and microarray analyses revealed increased expression of MERV-L when Kdm1a was mutated [89]. Interestingly, some cellular genes were also highly reactivated in Kdm1a mutant ESCs and blastocyst cells. Most of them harbored an LTR-derived sequence within two kilobases of their transcriptional start sites [89].

### 2.6. Histone Chaperones and Histone Variant H3.3 and Their Role in ERV Silencing

In addition to chromatin modifiers and repressor proteins, histone chaperones were found in a systematic genome-wide siRNA screen to induce proviral silencing in mouse ESCs [74]. Briefly, histone chaperones bind specifically to histones and deposit them onto DNA. They fulfill crucial functions to form and organize nucleosome formation during replication and/or replication-independent events such as DNA transcription or DNA repair [90]. The chromatin assembly factor 1 (Chaf1a) is a histone chaperone specific for histones H3 and H4, and was identified in an siRNA screen as a player mediating repression of class III ERVs through interaction with Kdm1a and Hdac2 as well as repression of class I and II ERVs by engaging Trim28 [74]. In one study, Chaf1a was shown to interact with HP1 via a specific motif at its N-terminus [70,91], and in another study was found to interact with both HP1 and Setdb1 (Figure 2) [74]. These interaction partners of Chaf1a induce heterochromatin formation by H3K9me3 formation and by reducing acquisition of transcriptionally active marks such as H3K4me3 or H3ac [74]. This study also showed that Asf1a and Asf1b double knock-down relieved the silencing effect comparable to the effect seen in Chaf1a knock-down cells. The Asf1 histone chaperones are also specific for H3 and H4 histone proteins and function both during replication-coupled and replication-independent nucleosome formation [90]. The authors proposed that the nucleosome assembly function of the two Asf1 isoforms, Asf1a and Asf1b, might be responsible for localizing Chaf1a to viral sequences [74].

Distinct sets of histone chaperones may be involved in loading distinct histone variants onto ERV DNAs. One study reported that the H3 histone variant H3.3 was enriched at class I and class II ERVs including IAPs and the histone chaperones Daxx and Atrx were found to perform H3.3 deposition onto ERV sequences [92]. In this study, the depletion of H3.3 led to reduced levels of H3K9me3 at ERVs and a dysregulation of nearby cellular genes [92,93,94]. In general, the histone variant H3.3 has not been described to play a consistently positive or negative role in cellular gene regulation. H3.3 has been linked to both active chromatin states, as defined by decoration with H3K4me3, H3K27ac, and H3K4me1, and alternatively with heterochromatin states, as defined by enrichment with H3K9me3, H3K27me3, and H4K20me3 [92,95]. A ChIP sequencing analysis revealed Daxx and Atrx occupancy on class I and II ERVs correlated with enrichment in Trim28. Daxx was shown to co-immunoprecipitate with Trim28, suggesting that it might be targeted to silence ERVs for H3.3 deposition. Furthermore, H3.3 histones were shown to co-localize with Trim28 on repetitive elements in ESCs and contribute to H3K9me3 formation of these genomic regions [92]. When H3.3 was depleted, Daxx and Trim28 recruitment was reduced. The authors hypothesized that H3.3 might play a role in ERV silencing that cannot be compensated by canonical H3 isoforms [92]. Atrx has been linked initially to heterochromatin formation by Trim28 recruitment on ERVs in a shRNA screen [96]. However, ES cells without Atrx, Daxx, or H3.3 revealed only a minor impact on ERV silencing, which makes it unlikely that these factors alone have an essential role in ERV silencing [92,96]. Further studies are needed to investigate the bigger picture.

### 2.7. DNA Methylation as a Mechanism to Silence ERVs

In addition to histone-based silencing, ERVs exhibit distinctive DNA methylation patterns. ERVs undergo *de novo* DNA methylation within the first days in mammalian embryogenesis [97]. Interestingly, *de novo* DNA methylation seems to require KRAB-Zfp as well as both Trim28 and Setdb1 binding [98]. The authors proposed that these interactions install stable epigenetic marks at ERV sites, which are subsequently maintained throughout development. Trim28 might provide a global protection of the genome from disadvantageous transcriptional dynamics during early embryogenesis. To investigate the role of DNA methylation on ERV silencing, DNA methyltransferase knock-out mouse models were generated to explore their role in regulating TEs. Three DNA methyltransferases in mammals (Dnmt1, Dnmt3a, and Dnmt3b) have been studied in detail. Dnmt3a and Dnmt3b function early in murine development as well as in germ cells and perform *de novo* DNA methylation. Dnmt3a and Dnmt3b expression in ESCs is very high, in contrast to very low expression levels in somatic cells [35]. Interestingly, a correlation between *de novo* DNA methylation through Dnmt3a and Dnmt3b and preexisting H3K9 methylation has been noted [99]. Dnmt3l is a catalytically inactive Dnmt3 homologue, but positively regulates the enzymatic activity of Dnmt3a and Dnmt3b [35,100]. In the context of ERV regulation, depletion of Dnmt3l and increased IAP expression in testis led to infertility due to loss of germ cells [101]. Dnmt1 was found to ensure maintenance of the DNA methylation signature. The lack of Dnmt1 led to a general transient ERV derepression in mouse ESCs [6,102,103], and Dnmt1 knock-out in somatic cells led to enhanced IAP expression. During mouse development, the lack of Dnmt1 caused termination of embryogenesis [102,104]. ESCs lacking either Dnmt3a or Dnmt3b alone showed normal methylation activity of endogenous as well as exogenous retroviruses [105]. However, double knock-down of Dnmt3a and Dnmt3b led to severe embryonic phenotypes similar to Dnmt1 knock-down [35]. Knock-out of all three DNA methyltransferases showed a complete loss of DNA methylation on ERV sequences, but only minor reactivation of transcriptional activity [6,40]. The “dogma” that ERV silencing can be divided into two mechanisms— first through Setdb1 and Trim28-mediated silencing via H3K9me3 in ESCs, followed by DNA methylation-mediated silencing in differentiated cells—has become less clear over time. The importance of DNA methylation seems to be very cell type-dependent [41,42,45,72,106]. It is interesting to note that a study recently proposed a correlation between the mechanism of silencing and the evolutionary age of ERVs [107]. CpG-rich young LTRs were found to be suppressed by DNA methylation, whereas intermediate age LTRs were predominantly silenced through posttranslational histone modifications such as H3K9me3 [107].

### 2.8. RNA-Mediated Targeting of TEs

In addition to the broad and diverse silencing mechanisms operating through DNA-specific binding by co-repressors and chromatin remodeling factors summarized here, another field is emerging: the RNA-mediated targeting of TEs. Mechanisms such as siRNA- or antisense transcripts-based silencing pathways have been reported to suppress IAPs as well as non-LTR retrotransposons such as LINE-1 [108,109]. Probably the most prominent phenomenon of RNA-dependent gene silencing is the X chromosome inactivation mediated by the long noncoding RNA Xist [110,111]. Notably, an important binding partner of Xist is Spen [112,113,114,115]. Recently, it has been shown that depletion of Spen reactivates a subset of ERVs in mouse ESCs. Spen has been found to bind to ERV-derived RNA and recruited several chromatin remodeling factors such as histone deacetylases [116]. Another example for RNA-mediated regulation of ERVs is the Piwi-interacting RNA (piRNA) pathway. Piwi proteins bind piRNAs, which degrade retrotransposon-derived mRNAs. In addition, piRNAs can induce chromatin changes and DNA methylation [117]. First discovered in Drosophila, piRNA has been reported to induce silencing through H3K9me3 formation [118]. In mice, knock-out of Piwi proteins led to derepression of IAPs and LINE-1 [119,120]. Piwi proteins are especially expressed in germ cells to protect the genome from transposition events. The precursor molecules of piRNAs are organized in so called piRNA clusters, often enriched at TEs [35].

## 3. Transcriptional Regulation of Human ERVs

### 3.1. Nomenclature and Expression of Human ERVs

About 8% of the human genome is comprised of TEs containing LTRs. The majority of these elements have lost various regions of similarity to retroviral coding gene sequences due to massive accumulation of mutations over the course of evolution. In contrast to mouse, all ERVs extant in the human genome are replication-defective, with HERV-K (HML-2) most likely the element that is closest to being replication competent [121]. Many ERVs consist of only a solitary LTR, likely formed by homologous recombination between the two LTRs of a parental provirus. However, around 40 families still retain recognizable sequence similarity to coding regions of proviruses. In one nomenclature scheme, the human ERV families (HERVs) have been named on the basis of the specific cellular tRNA matching the PBS of the element, and presumably used by the HERV reverse transcriptase to initiate DNA synthesis [8,122]. For example, the youngest HERV family so far identified in the human genome has a lysine tRNA PBS and thus was named HERV-K. An overview of selected members of the three HERV classes are depicted in Figure 1C.

A comprehensive microarray study determined the transcription profiles of HERVs in healthy human tissues [123]. Importantly, all of the 19 tested tissues showed HERV transcripts, confirming that HERVs are far more than “retroviral fossils” and are actively expressed in a variety of cell types. The HERVs are differentially expressed in a cell type-specific manner, similar to common genes. In general, class I and class II HERVs seemed to be expressed more frequently than members of class III. The majority of class I and class II HERVs were established about 40 to 50 million years ago and the younger members of class II approximately 5 million years ago, whereas class III HERVS are at least 70 million years old. The less frequent expression of the class III HERVs might be explained by the fact that older elements have had a longer time to acquire more mutations, and to have been selectively removed from gene-rich regions to maintain genomic stability [123]. Interestingly, there was evidence that HERV activity might correlate with the transcriptional and proliferative rate of a cell, since thyroid glands, skin, reproductive organ tissues, and tissues of embryonic origin showed HERV expression of all HERV classes, whereas terminally differentiated, non-dividing muscle cells revealed less HERV activity in general [123].

### 3.2. Transcriptional Regulation of Human ERVs

It became clear that our “viral fossils” have undergone a long history of co-evolution with our genome. Notably, 25% of regulatory regions in the human promoter database harbor TE-derived sequences [124]. Of course, this does not necessarily mean that all of them possess biological relevance. However, the question remains of how their transcription and their adverse effects are managed and how genomic stability is maintained over millions of years. A very common mechanism to ensure silencing of ERV sequences in human is through KRAB-ZFPs, and as described in mice above, through TRIM28, SETDB1, and DNA methyltransferases. Large scale DNA-binding analyses identified that two thirds of human KRAB-ZFPs bind to TEs in a direct and specific manner [44,125,126,127]. TRIM28 is expressed in most cell types, but it is especially highly expressed early in embryonic development. In addition to its role in the silencing of TEs, TRIM28 depletion has been described to upregulate interferon-stimulated genes. Moreover, the authors showed activation of MAVS-dependent innate immune responses after TRIM28 depletion [128]. A more recently discovered silencing mechanism of HERVs is through Tat-interactive protein 60 (TIP60), a lysine acetyltransferase. Together with the chromatin reader protein BRD4, TIP60 positively interacts with the H3K9 methyltransferases SUV39H1 and SETDB1 to establish global H3K9me3 levels [129]. Depletion of TIP60 in colorectal cancer cells led to a genome-wide TE reactivation [129].

### 3.3. Deregulation of Human ERVs and Diseases

Although most HERVs have lost the ability to carry out retrotransposition and create new insertional mutations, they still influence host cell signaling and gene regulation either through their viral mRNA or viral protein products, or their remaining LTR-derived gene regulatory regions, which can affect genes even at a considerable distance [130,131]. Additionally, the existence of multiple copies increases the likelihood of recombination events between pairs of elements, leading to large inversions or deletions. These circumstances might explain how HERVs possess the potential to influence disease progression. Elevated levels of ERV-derived mRNA or proteins together with ERV-specific antibodies have been measured in certain diseases. Various studies suggest that transcriptional expression of certain TEs to be involved in neurological disorders such as Rett syndrome, amyotrophic lateral sclerosis (ALS), schizophrenia, autistic spectrum disorder (ASD) or multiple sclerosis (MS) [132,133,134,135]. In the case of the autoimmune disease ALS, elevated HERV-W envelope protein expression was detected in muscle cells of ALS patients [136]. In addition, HERV-K expression was found to be elevated in autopsy brain tissue of ALS patients. HERV-K envelope products were detected in cortical and spinal neurons, which show motor dysfunction, suggesting a correlation of HERV-K with neurodegeneration [137,138]. HERV-K has even qualified for the use as a genomic marker for ALS. MS is another example for an autoimmune disease, where HERV expression levels exhibit an elevated abundance. HERV-W was reported to be highly expressed in neural plaques of MS patients. The HERV-W envelope has been detected in the brain, especially in brain lesions of MS patients in contrast to healthy controls [139]. Moreover, the HERV-W envelope has been shown to contribute to the neurodegenerative phenotype of MS [140,141]. Importantly, monoclonal neutralizing antibodies against the HERV-W envelope are being used in clinical trials as a neuroprotective treatment for MS [122,140]. In the case of the neurodevelopmental disorder ASD, HERV-H and HERV-W were identified to exhibit differential expression patterns in patients compared to healthy controls [135]. HERV-H showed higher abundance, whereas HERV-W showed less abundance in ASD blood samples compared to healthy control samples [135]. Furthermore, HERV-H was expressed at significantly higher levels in ASD patients with severe disease development. ERV expression patterns might have the potential to serve as a useful biomarker for ASD, especially important since there is the need for reliable markers for this disease [142]. HERVs may well serve as regulatory elements in the brain and imbalance might contribute to a specific disease phenotype [44].

Aside from neurological disorders, deregulated expression of HERVs in various cancer cell types has been reported. HERV-K expression is an example that has been linked to many cancers such as breast cancer, lung cancer, prostate cancer, melanoma, germ cell tumor, lymphoma [122,138,143]. The question is still unresolved as to whether ERVs play a causative role or whether their upregulated expression is a consequence of the pathogenic phenotype of the cell and, for example, a result of global hypomethylation [8]. The direct connection of ERV expression and disease remain controversial.

### 3.4. Human ERVs and Their Upregulation through Exogenous Viruses

HERV transactivation has been reported to be triggered by infections of a number of human exogenous viruses such as HIV-1, hepatitis B and C, human T-lymphotropic tumor virus-1 (HTLV-1), influenza A or herpes virus [130,144]. In the case of HIV-1, a study demonstrated that recombinant HIV-1 Tat protein increased HERV-K (HML-2) Gag RNA expression substantially in primary lymphocytes and that this upregulation involved transcription factors NF-κB or NF-AT [145]. These data indicate that exogenous virus infection activates transcription factors, which consequently also bind to HERV LTR regions and induce their transactivation. Moreover, a study observed that in different human packaging cell lines for retroviral vector systems, utilized for HIV or MLV production, packaged viral particles always contained contamination by cellular vesicles or exosomes, which also contain HERV transcripts [146]. These data demonstrate that exogenous virus infection can serve as carriers of HERV products for transfer into different target cells.

Interestingly, envelope proteins from the HERV-H and HERV-K family were shown to have immunosuppressive functions in vivo. This observation reveals that HERVs possess the potential to regulate the host immune system [147,148]. This might be to the benefit of the HERVs, but which is also exploited by their exogenous relatives to prevent antiviral immune activity. Consequently, a better understanding of how TEs are transactivated through exogenous viruses will help to find novel targets for virus-mediated diseases or virus-mediated tumor treatment.

## 4. Co-Option of ERV Functions for the Benefit of the Host

There is an increasing number of examples in which TEs were domesticated for the benefit of the host. This process, in which the host makes use of TE-derived functions, are often called exaptation, co-option, or simply repurposing. Either regulatory elements or even encoded proteins, can be beneficial to the host. TE sequences can serve as alternative promoters, enhancer elements, alternative splice sites, or polyadenylation signals [22,149,150,151,152,153]. TE sequences also reveal the potential to serve as a hub for DNA-binding transcription factors, and their binding can influence nearby host genes. Since RNA transcripts are crucial intermediates of the ERV life cycle, the elements often contain transcriptional activators in their LTR regions, which regulate the expression of the ERV but can also impact transcription of nearby host genes even over considerable distances [143]. Most ERV sequences are neutral and not detrimental for the host, but deregulation of host genes can lead to adverse events for the host, as described above. However, ERVs can alter host gene regulation in a beneficial manner. Regulatory elements of the ERVs are perhaps the most commonly utilized sequences, either in profoundly controlling host genes, or for fine-tuning of gene expression, providing fitness advantages during evolutionary development [154]. In one example, a study revealed that one third of the protein binding sites of transcription factor p53 are enriched at ERV sequences [155]. This factor, with many pleiotropic functions, is one of the most important master regulators of gene expression in the cell. Evolutionary analysis of the study dated the appearance of ERV-containing p53 binding sites at around 40 million years before the present [155]. The authors hypothesized that p53 sites arose and were altered on ERVs, and that these “viral fossil sequences” contributed substantially to the evolution of the host genome over tens of millions of years. In a similar way, a ChIP-sequencing analysis revealed that interferon gamma (IFNγ)-inducible transcription factor binding sites were enriched in 27 TE families, from both young and ancient ERVs [156]. Binding sites for inflammatory transcription factors, such as NF-κB or members of the IRF family, have been reported [157,158]. These findings give evidence that ERV functions were co-opted to regulate host genes involved in innate immunity. They also suggest that the functions of the ERV LTRs to regulate the immune system provide an ancient record of the arms race between virus and host—a mechanism by which the virus supports its transcription and replication [156,159]. An example for an ERV gene product that has the potential to modulate the innate immunity is the HERV-K accessory protein Rec. Rec has been shown to elevate IFITM1 levels, which induces restriction of viral entry [160]. This mechanism serves as a protective mechanism against invading viruses during early development. Various HERVs have been found to exhibit distinctive complex patterns of expression across early development. For example, HERV-H elements are preferentially expressed in ESCs and at very early stages of embryogenesis [161,162]. As HERV-H sequences contain binding sites for several pluripotency transcription factors, and because HERV-H RNAs are very abundant in human pluripotent stem cells, it has been suggested that HERV-H plays a role in pluripotency [163]. Interestingly, HERV-H expression was increased in the primed state, as compared to the naïve pluripotency state [164]. Furthermore, a recent study demonstrated that HERV-H creates topologically associating domains (TAD), with which they directly influence the cell-type-specific chromatin landscape [165]. Characterizing the naïve pluripotency state of human stem cells is challenging, but important, because a deeper understanding of this unique pluripotency feature would offer immense potential for gene therapy approaches [166]. In addition to HERV-H, elevated RNA and protein expression of HERV-K such as viral-like particles and Gag proteins were observed in human blastocysts [160,167]. Differentiation of pluripotent cells led to a downregulation of HERV-K, accordingly, HERV-K is used as a validated pluripotency marker [167].

Another regulatory network that likely functioned as a driving force in ERV silencing in humans, as in mice, is the arms race between KRAB-ZFPs and TEs [168]. ZFPs are the largest family of transcription factors and the human genome itself encodes more than 350 KRAB-ZFPs [44,125]. Several KRAB-ZFPs are human-specific due to their evolution during the primate linage. Many ZFPs act to silence HERVs, in particular cell types of specific developmental stages. Although mammal genomes share similarities regarding their TE sequences, many TEs are species-specific, leading to species-specific differences in their TE-influenced gene regulatory networks. KRAB-ZFPs exhibit an abundant expression profile in all human tissues and many are highly expressed in the brain [125]. It has been speculated that TEs might have driven a more rapid evolution of primate and human gene regulation in the brain, and might have contributed to differences in complex cognitive functions [44]. These ideas seem wild, but the field of ERV function in regulating host gene expression has just started to be explored, and more support for this theory may soon be uncovered. Although many ZFPs are known for their ability to silence TEs, other KRAB-ZFPs silence host genes rather than viral genes. Repressed expression of placental-specific insulin-like growth factor 2 (Igf2), an important fetal growth hormone, has been linked to Zfp568 in mice [169].

ERV proteins, encoded by viral open reading frames, have also been exapted for the benefit of the host. A prominent and powerful example of exaptation of ERV envelope glycoproteins are the ERV-encoded syncytins. These proteins, used by viruses to mediate virus–cell membrane fusion, were co-opted for cell–cell fusion, and now function in placenta formation in mammals. The syncytins induce formation of the multinuclear syncytiotrophoblast through receptor-mediated membrane fusion [8,170]. Notably, exaptation events of syncytins are thought to have evolved independently across the different branches of placental mammalian lineages [8]. Notably, placenta fusion events during human embryogenesis are driven by syncytin1, derived from HERV-W, and syncytin2, encoded by HERV-FRD [171].

Another fascinating example of co-option of HERV envelopes is called superinfection interference. Previously endogenized exogenous retroviruses confer resistance to further viral infections [8]. The murine *Friend virus susceptibility 4* (*Fv4)* gene, an incomplete remnant of an MLV provirus, acts as a restriction factor to prevent exogenous infection by ecotropic MLV at the cellular entry step of the retroviral life cycle [8,172,173]. A different example of an ERV-based restriction factor is Friend virus susceptibility 1 (Fv1), which antagonizes certain classes of MLVs. Fv1 originated from a viral *Gag* gene with similarities to the ORFs in HERV-L or MERV-L in mouse [174,175]. Fv1 functions after cellular entry, but before provirus formation [176].

More recently, the co-option of an ERV Gag protein has been explored. Activity-regulated cytoskeleton-associated (ARC) proteins generate virus-like particles, perform intercellular mRNA transfer in neurons and fulfill important functions in neuronal development [177,178,179]. ARC proteins were derived from a Ty3/Gypsy retrotransposon *Gag*, and its functions of self-assembly and encapsidation of nucleic acids have been repurposed for the benefit of the host [177].

Many efforts are underway to make use of the induction of ERV expression for cancer treatment. Drug-dependent derepression of HERVs induces a state of cell activation by a process sometimes called viral mimicry. Reactivated HERVs generate double-stranded RNA products, which activate type I or type III interferon responses, resembling the antiviral response upon viral infection [180]. HERV-encoded peptides can also serve as neoantigens to induce immune rejection of cells induced to derepress HERVs. The induction of viral mimicry leads consequently to immunogenic cell death of cancerous cells [181,182]. Clinical trials using DNA demethylating agents and HDAC-inhibitors are ongoing to target multiple cancer types in human such as colorectal cancer, ovarian cancer, promyelocytic leukemia, or hepatocellular carcinoma [180]. As described above, several cancer types exhibit overexpression of HERV proteins, like HERV-K. Targeting of ERV-derived so called tumor-associated antigens can be exploited to develop, for example, cancer-specific chimeric antigen receptor (CAR) T-cell therapy strategies as well as the development of effective vaccine approaches [180]. These examples show that although HERVs are, on one hand, thought to promote pathological progression of various cancers, neurological disorders, or autoimmune disease, they can on the other hand be used to contribute to help to visualize and target otherwise immunologically invisible cancer cells.

## 5. Concluding Remarks

Why do ERVs persist in our genome over millions of years? How do humans function with almost half of the genome derived from transposable sequences? There are still more questions than definite answers with regard to our ancient “roommates”. Perhaps they first entered our genomes selfishly as successful parasites. They were then silenced by our defense systems, and then domesticated for the benefit of the host in response to both negative and positive evolutionary forces to avoid severe genomic instability and to employ the potential for transcriptional gene regulation, new binding sources for transcription factors, and even exaptation of viral genes. While many of the players in ERV silencing have been identified, more are yet to be found, and the detailed mechanisms for ERV silencing are still not fully understood. It is not clear why some species or even individuals show higher endogenous ERV activity than others. To fully understand transcriptional silencing of ERVs we will need to understand the mechanisms for the establishment of certain DNA- or histone-based posttranslational modifications as well as the maintenance and re-establishment of these silencing marks during replication and DNA damage repair. TRIM28 is unquestionably a master regulator for transcriptional silencing of many retroviruses, both exogenous and endogenous. Nevertheless, the full story of ERV expression and regulation is still not complete and other regulators and contributing factors remain to be identified. Finally, there are specific settings of enormous research interest in which ERVs were found to be deregulated, for example, in aging processes and memory impairment [183,184,185,186]. Are ERVs the keys to understanding the mysteries of aging? Time will tell.
